# Fast and Simple UPLC–Q-TOF MS Method for Determination of Bitter Flavan-3-ols and Oligomeric Proanthocyanidins: Impact of Vegetable Protein Fining Agents on Red Wine Composition

**DOI:** 10.3390/foods12173313

**Published:** 2023-09-02

**Authors:** Lara Dias, Juliana Milheiro, Miguel Ribeiro, Cristina Fernandes, Nuno Neves, Luís Filipe-Ribeiro, Fernanda Cosme, Fernando M. Nunes

**Affiliations:** 1 Chemistry Research Centre-Vila Real (CQ-VR), Food and Wine Chemistry Laboratory, University of Trás-os-Montes and Alto Douro, 5000-801 Vila Real, Portugal; lara.dias96@outlook.com (L.D.); juliana.milheiro@hotmail.com (J.M.); jmribeiro@utad.pt (M.R.); fmota@utad.pt (L.F.-R.); 2 Genetics and Biotechnology Department, School of Life Sciences and Environment, University of Trás-os-Montes and Alto Douro, 5000-801 Vila Real, Portugal; 3 Sogrape Vinhos S.A., 4430-809 Avintes, Portugal; cristina.fernandes@sogrape.pt (C.F.); nunomiguel_neves@hotmail.com (N.N.); 4 Biology and Environment Department, School of Life Sciences and Environment, University of Trás-os-Montes and Alto Douro, 5000-801 Vila Real, Portugal; 5 Chemistry Department, School of Life Sciences and Environment, University of Trás-os-Montes and Alto Douro, 5000-801 Vila Real, Portugal

**Keywords:** wine, proanthocyanidins, UPLC-MS, wine fining, protein fining agents, animal protein, vegetable protein

## Abstract

Wine phenolic compounds, particularly proanthocyanidins (PAs), play a significant role in wine sensory characteristics, specifically bitterness and astringency. Although not consensual, flavan-3-ols and oligomeric PAs are generally considered the primary contributors to wine bitterness. Patatin, a vegetable protein fining agent, has been explored as an alternative to animal and synthetic fining agents for reducing wine bitterness. However, contradictory results exist regarding its effectiveness in removing flavan-3-ols and oligomeric PAs in red wines. In this work, a UPLC–Q-TOF MS/MS method was optimized and validated for accurately measuring flavan-3-ols, as well as dimeric and trimeric PAs, in red wines. The MS/MS analysis of flavan-3-ols, in addition to the typical fragmentation described in the literature, revealed an intense mass fragment resulting from the loss of C_3_O_2_ and C_3_O_2_ + H_2_O from the parent ion. It was observed that flavan-3-ols and PAs undergo oxidation during sample preparation, which was reversed by the addition of 5 g/L of ascorbic acid. The method demonstrated good linearity range (2 mg/L to 20 mg/L), detection limit (0.3 mg/L to 0.7 mg/L), quantification limit (0.8 mg/L to 2.2 mg/L), precision (repeatability 2.2% to 7.3%), and accuracy (recovery 98.5% to 100.5%). The application of patatin at different doses (5 g/L to 30 g/L) in two different red wine matrices did not reduce the levels of monomeric, dimeric, and trimeric PAs in red wines. However, similar behaviors were observed for pea protein and gelatin. Therefore, wine fining trials and efficiency measurements of the treatments in each matrix are strongly advised.

## 1. Introduction

The perception of wine quality is primarily influenced by the absence of any defective aromas. Additionally, it is shaped by the presence of non-volatile components, particularly the phenolic composition, which can significantly impact the perception of quality [1]. Monomeric, oligomeric, and polymeric flavan-3-ols are responsible for several positive and negative sensory properties of wines, such as astringency and bitterness. Proanthocyanidins (PAs) consist of flavan-3-ol subunits with a degree of polymerization (DP) equal to or greater than 2, mainly linked by (4 → 8) or (4 → 6) carbon–carbon bonds (B-type). In some botanical sources, an additional (2 → 7) ether linkage also occurs (A-type). Depending on the type of monomers, proanthocyanidins can be classified into procyanidins, composed of (+)-catechin and (−)-epicatechin monomers, prodelphinidins, composed of (−)-gallocatechin/(−)-epigallocatechin monomers, and propelargonidins, composed of (+)-afzelechin/(−)-epiafzelechin monomers. In certain positions, proanthocyanidins may sometimes be esterified with gallic acid or exceptionally with sugars [2]. Wine bitterness has mainly been attributed to the presence of small-molecular-weight flavan-3-ols, especially epicatechin, as well as dimeric and trimeric PAs. Catechin, albeit to a lesser extent, also contributes to bitterness [3,4,5,6,7]. High-molecular-weight PAs are responsible for astringency [8,9,10]. Although the levels of low-molecular-weight PAs in many wines are below their sensory threshold [3,5,11], their presence has been shown to act synergistically [12].

Astringency and bitterness are two sensory characteristics of wines that can be managed using protein fining agents, as well as the synthetic polyvinylpyrrolidone (PVPP) polymer [13,14]. Each fining agent has its own selectivity in removing specific phenolic fractions in wines, such as flavan-3-ols, oligomeric PAs, and polymeric PAs. These fining agents are widely used in the wine industry. Nevertheless, vegetable-based protein fining agents are gaining popularity due to their lack of allergenicity, sustainability, and appeal to the vegan market [13,14].

Among the alternative vegetable-based protein fining products available on the market are potato proteins. Potatoes contain an active protein called patatin, which constitutes 40% of the total soluble potato protein and is obtained from an aqueous by-product of potatoes [15]. Patatin’s molecular weight ranges from 15 kDa to 120 kDa, with the majority being around 40 kDa [16]. The patatin protein has a pI of 4.6 and low solubility at wine pH [17], making it a low-risk option for over-fining [18]. Potato proteins have demonstrated a good capacity to fine wine phenolics and reduce grape must turbidity [18,19,20,21,22]. In terms of fining efficiency, potato proteins have been shown to be comparable to gelatin in terms of phenolic removal and reduction of astringency sensation in commercial and model wine with added grape seed extract. They have also been found to be more effective than other traditional fining agents, such as casein, egg albumin, PVPP, and vegetable-derived protein fining agents, such as pea, soybean, and rice [18,22]. While potato protein fining does not significantly affect overall flavor intensity and bitterness, it can influence wine color intensity and hue [22].

For proteinaceous fining agents, significant variability has been shown in their selectivity for PA molecular size classes based on the characteristics of the protein [23]. The potential to modulate wine PA composition is an important aspect when using fining agents, in addition to reducing PA concentration. However, studies attempting to draw conclusions about the selectivity of proteinaceous fining agents toward PAs have produced diverse results. Early research demonstrated that proteins preferentially bind to PAs with higher molecular weight and higher galloylation degree [24,25]. Subsequent studies revealed that the selectivity of commercial proteins for PAs is determined by the composition of the protein and its molecular weight [23,26,27]. Additionally, the fining effect is strongly influenced by factors such as the wine matrix, protein dose, protein composition, and polydispersion.

Proteins from peas have also been tested for their fining efficiency compared to commercial standards such as k-caseinate and gelatins [28]. These studies showed that pea proteins are as effective as gelatin in fining. However, the fining ability of pea proteins can be significantly modified by limited protein hydrolysis. In another study, it was demonstrated that pea proteins have a similar ability to remove tannins and reduce astringency compared to PVPP, but they were less effective than gelatin [22]. Researchers have also investigated the potential of insoluble protein isolates from peas as fining agents in model wine and white wine [29]. Pea proteins were found to reduce wine aroma similarly to commercial fining agents. A comparison of pea proteins, PVPP, and k-caseinate for white wine fining was conducted [30]. The results indicated that both flavonoid and non-flavonoid phenols significantly decreased in all wines treated with the three fining agents, as well as the wine color. Only k-caseinate, however, reduced the wine browning potential. Pea protein and k-caseinate were considered the most effective for wine clarification, while, from a sensory perspective, all fining agents had similar effects on the wine.

Despite the importance of monomeric and oligomeric PAs from a sensory, technological, and health point of view, their quantification in red wines poses several challenges, particularly for aged red wines and fortified sweet wines. Both normal-phase and reversed-phase high-performance liquid chromatography (HPLC) have been employed in the analysis of PAs. The choice of chromatographic method depends on the types of PAs expected in the sample and the research objectives [31]. In general, normal-phase HPLC is used for separating high-molecular-weight PAs, as their hydrophilicity increases with increasing molecular weight, while reversed-phase HPLC is typically employed for separating oligomeric PAs and low-molecular-weight isomers [32,33]. Diode array detection (DAD) is commonly used for the detection of PAs in wine due to its high sensitivity and ease of operation. However, the identification and quantification of PAs in wine can be complex due to the sample matrix’s complexity and the low concentration at which they are present. Additionally, the UV spectra of PAs are very similar, which can lead to ambiguous identification. Furthermore, although monomeric and oligomeric PAs exhibit good retention on reversed-phase columns, their maximum absorption occurs at approximately 277 nm, and they have lower extinction coefficients compared to anthocyanins and cinnamic acid derivatives. Given these challenges, a more sensitive and selective detector is often necessary. Currently, liquid chromatography/mass spectrometry (LC–MS) using tandem mass spectrometry is considered the best analytical technique for studying phenolic compounds in grapes and wines [33,34,35]. Multiple types of mass spectrometers can be used for polyphenol analysis, such as triple quadrupole [36,37], ion trap [38,39,40], time-of flight or quadrupole-time-of-flight [41,42,43], and Orbitrap [44]. The hybrid quadrupole time-of-flight (Q-TOF) mass analyzer provides excellent mass accuracy over a wide dynamic range, and measurements of the true isotope pattern elucidates the molecular formula of unknown metabolites with a high degree of reliability [43]. It also performs tandem MS, which provides more detailed structural information, especially when standard compounds are not available. Furthermore, the high mass accuracy of Q-TOF provides high selectivity in the extracted-ion chromatogram (EIC) mode, particularly when there are overlapping peaks, which could be problematic in spectrophotometric detection. Thus, HPLC coupled to highly sensitive and high-resolution MS, such as Q-TOF, enables the separation and detection of minor compounds that could co-elute with major ions and be underestimated, unidentified, or even not detected by older methodologies [45,46]. The Q-TOF configuration allows collimation of the ion beam, as well as the option of mass selection in Q1 and fragmentation in a radiofrequency-only quadrupole. They can produce high-quality MS/MS spectra, including high-resolution data for the determination of molecular formulae. Both MS and MS/MS experiments can be performed for high-accuracy and high-resolution analysis. Additionally, multiple reaction monitoring (MRM) detection has a few advantages compared to selected ion monitoring (SIM), such as much higher selectivity with less interference of co-eluting compounds and matrix components, and a better signal-to-noise ratio, allowing quantitation with lower limits of quantitation (LOQ) [47]. Moreover, fewer ions must be detected per compound in MS/MS in comparison to MS for confirmatory analysis [48]. Furthermore, a wider linear range, higher accuracy, and reproducibility can be obtained.

Therefore, the purpose of this work was to evaluate the fining efficiency of vegetable proteins available on the market, namely, patatin and pea protein, and compare their efficiency with the traditional animal-based fining agents in removing potential bitter monomeric, dimeric, and trimeric flavan-3-ols. To achieve this, we optimized and validated a fast, accurate, and precise LC–Q-TOF MS/MS method for quantifying the levels of monomeric, dimeric, and trimeric flavan-3-ols present in wine.

## 2. Materials and Methods

### 2.1. Reagents and Chemicals

(−)-Epicatechin and (+)-catechin standards, and ascorbic acid were provided by Sigma Aldrich. (−)-Epigallocatechin, (−)-epigallocatechin gallate, (−)-epicatechin gallate, procyanidin B1, procyanidin B2, and procyanidin C1 standards were purchased from Extrasynthese (Genay, France). Wine samples used in this study were provided by Sogrape Vinhos, SA. The eluents used in the LC–MS analysis were methanol and acetic acid (CH_3_COOH) at a concentration of 0.1%, both provided by Sigma Aldrich (St. Louis, MO, USA). Ultra-pure deionized water was obtained from a Milli-Q water purification system. Gelatin was supplied from Proenol (Vila Nova de Gaia, Portugal), and vegetable proteins were supplied by Agrovin (Ciudad Real, Spain) and SAI Enology (Penafiel, Portugal). One patatin and one pea protein from each manufacturer were used. Producers guaranteed that proteins were not from genetically modified organisms (GMOs).

### 2.2. Sample Preparation

For wine analysis, the samples were previously diluted 20 times with distilled water, and ascorbic acid was added to a final concentration of 5 g/L. Samples were filtered through a regenerated cellulose 0.45 μm filter and placed in a vial, and then stored at room temperature until analysis. For the quantification of red wine PAs, nine randomly selected samples were used. The main enological characteristics of these wines are presented in Supplementary Material Table S1.

### 2.3. Chromatographic Analysis

Liquid chromatography analyses were performed using a UPLC system (Dionex Ultimate 3000, Sunnyvale, CA, USA) equipped with an HPG-3400RS pump, a WPS-3000TRS automatic injection system, and a TCC-3000RS column oven. In turn, the detection was carried out using an MS maXis impact detector (Bruker, Karlsruhe, Germany) supplied with an ESI ionization source, a QTOF mass analyzer, and a microchannel plate detector. Acquisition was made in broadband collision-induced dissociation (bbCID) mode. The key principle of this technique is to simultaneously fragment all precursor ions previously detected in the MS survey scan. Alternating between MS survey scan (low energy) and bbCID scans (higher energy) enables the generation of MS scans for all precursor ions and their fragment ions across the mass range. Separation was carried out using a 2.2 μm 120 Å reversed-phase C18 column (Thermo Scientific, Waltham, MA, USA) at 30 °C. Solvents were (A) acetic acid 0.1% and (B) methanol at a flow rate of 0.3 mL/min. The sample injection volume was 2.0 µL, and the gradient used is presented in Table 1.

### 2.4. Method Validation

The proposed method was validated in accordance with the recommendations of the International Conference on Harmonization (ICH) [49] for dry red wine.

#### 2.4.1. Calibration Curve and Linearity

The linearity of the method was assessed for (+)-catechin, (−)-epicatechin, (−)-epigallocatechin, (−)-epigallocatechin gallate, (−)-epicatechin gallate, procyanidin B1, procyanidin B2, and procyanidin C1. Individual stock solutions of these compounds were prepared in methanol. These solutions were diluted and pooled to obtain a single standard solution at a concentration of 10 mg/L in deionized water containing 5 g/L ascorbic acid. Working solutions were prepared with a concentration ranging from 0.1 mg/L to 1.0 mg/L in red wine (Wine 1 from Alentejo’s region). Linearity was assessed by calculating the QCmean [50] and Mandel’s fitting test [51]. The idea of Mandel’s fitting test is to compare the standard error (Sy1) of a linear regression model with the standard error of the second-order polynomial regression model (Sy2). The value of F obtained is compared with the distribution of F with 1 and n − 3 degrees of freedom.

#### 2.4.2. Detection and Quantification Limits (LOD and LOQ)

The limit of detection (LOD) and the limit of quantification (LOQ) were calculated for each compound. These parameters were calculated using the ratio between the standard error of regression by the slope of a calibration curve up to 0.4 mg/L, which was performed in triplicate. The result was then multiplied by 3.3 (LOD) and 10 (LOQ), considering the dilution factor. In the proposed method, 20-fold dilution was used.

#### 2.4.3. Repeatability and Intermediate Precision

One red wine sample with a known amount of proanthocyanidins in the study was analyzed in triplicate on three different days. The three standard deviations of the three triplicates were combined to obtain the values of method repeatability. The intermediate repeatability was evaluated in triplicate in three different working sessions using the same procedure and the same sample. Repeatability and intermediate repeatability can be determined by calculating the residual standard deviation (RSD), expressed as a percentage. The predicted relative standard deviations of repeatability (PRSDR) and intermediate repeatability (PRSDIR) were calculated using the Horwitz formula, and the Horwitz ratio (HorRat) was calculated between the experimental relative standard deviation and that calculated from the Horwitz formula. The limit of acceptability for HorRat values is 0.5 to 2.0 [52].

#### 2.4.4. Recovery

The accuracy of the quantifications was determined by calculating the recoveries using the standard addition method. The recovery was determined by adding increasing concentrations of the analytes to a red wine sample and analyzing it three times. The recovery results for the flavan-3-ols, as well as dimeric and trimeric procyanidins, were obtained from spike and recover experiments, ranging from 0.10 mg/L to 1.0 mg/L (n = 6 × 3) for the 20-fold diluted wine. These concentrations corresponded to flavan-3-ols concentration in undiluted wine ranging from 2 to 20 mg/L.

### 2.5. Analytes Positive Identification

For positive identification of flavan-3-ols in red wine samples, the identification points (IP) system was used [48], with a threshold of IP > 4 considered acceptable. For catechin, epicatechin, epigallocatechin, procyanidin B1, procyanidin B2, and procyanidin C1, a threshold of 7.5 IPs was used, which involved the use of 3 high-resolution product ions. For epicatechin gallate and epigallocatechin gallate, a threshold of 5 IPs was used, involving the use of 2 high-resolution product ions. In addition, the ion ratios were measured and found to be within the recommended maximum permitted tolerances [48].

### 2.6. Analysis of Protein Fining Agents by Sodium Dodecyl Sulfate Polyacrylamide Gel Electrophoresis (SDS-PAGE)

The protein fining agents were diluted in a sample buffer consisting of 2% (*w/v*) sodium dodecyl sulfate (SDS), 40% (*v/v*) glycerol, 0.02% (*w/v*) bromophenol blue, and 0.08 mol/L Tris-HCl pH 8.0. The mixture was heated at 70 °C for 10 min and then separated using a polyacrylamide resolving gel. The resolving gel had a total monomer concentration (T) of 12.52% and a crosslinker weight percentage (C) of 0.97%, as described in a previous study [53]. Gel electrophoresis was conducted using a Hoefer SE 600 Ruby unit (Amersham Biosciences, Uppsala, Sweden) at a constant current of 30 mA/gel. Subsequently, the gels were stained with a solution containing 0.04% (*w/v*) Coomassie Blue R-250 and 9.6% (*w/v*) trichloroacetic acid (TCA) for 24 h, followed by overnight washing in distilled water.

### 2.7. Fining Experiments

The three fining agents were applied at a concentration of 50 g/hL. Liquid gelatin was directly added to the wine, while pea proteins and patatins were first dissolved in water before being added to the wine as suggested by the manufacturers. A control group was used consisting of wine without any enological products. The trials were conducted in 250 mL of wine, and the fining agents were thoroughly mixed and left in contact with the wines for 3 days at room temperature (approximately 21 °C). Two different red wines were used in the fining experiments, Wine 1 from Alentejo region and Wine 2 from the Douro region. The main enological characteristics of these wine samples are presented in Table 2.

### 2.8. Statistical Analysis

The data are presented as means ± standard deviation. Physicochemical data were statistically tested by analysis of variance (ANOVA) followed by the Tukey post hoc test (*p* < 0.05), using Statistica 7 software (Statsoft, OK, USA).

## 3. Results and Discussion

### 3.1. Method Development and Optimization

#### 3.1.1. Optimization of Tandem Mass Spectrometry Detection

**Figure 1:**
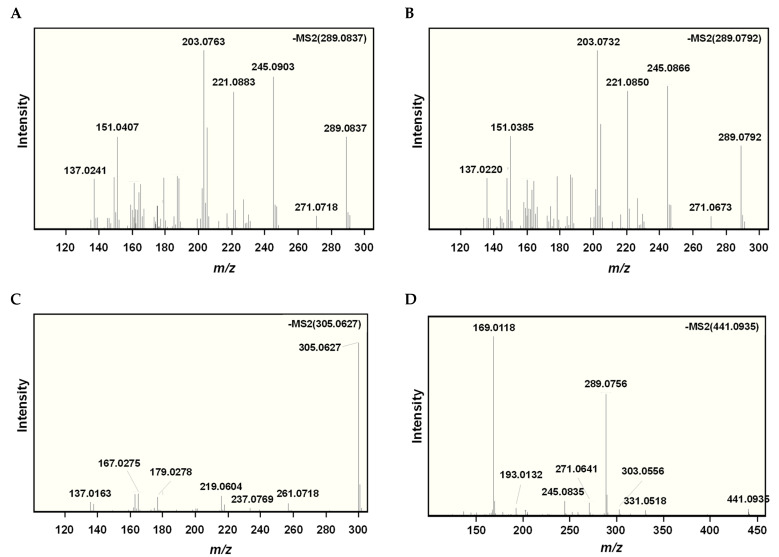

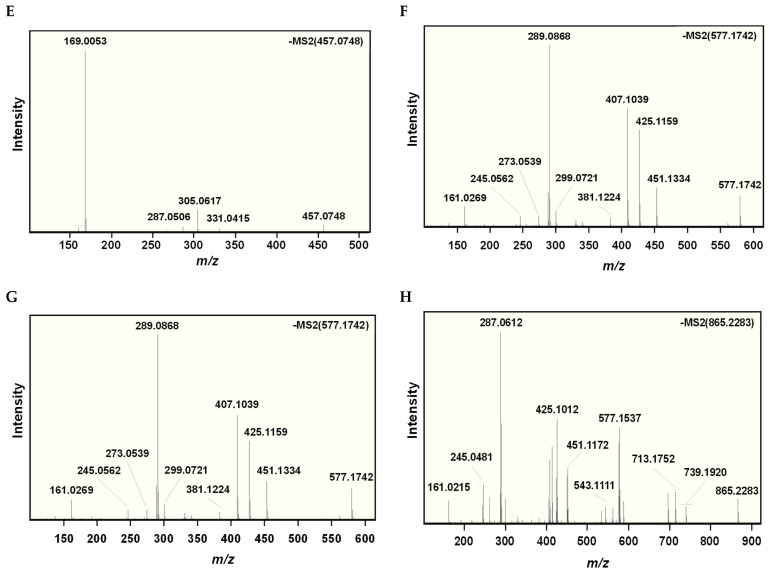
ESI/Q-TOF tandem mass product of flavan-3-ol monomers and B-type dimers and trimer standards of (**A**) catechin, (**B**) epicatechin, (**C**) epigallocatechin, (**D**) epicatechin gallate, (**E**) epigallocatechin gallate, (**F**) procyanidin B1, (**G**) procyanidin B2, and (**H**) procyanidin C1.

**Scheme 1:**
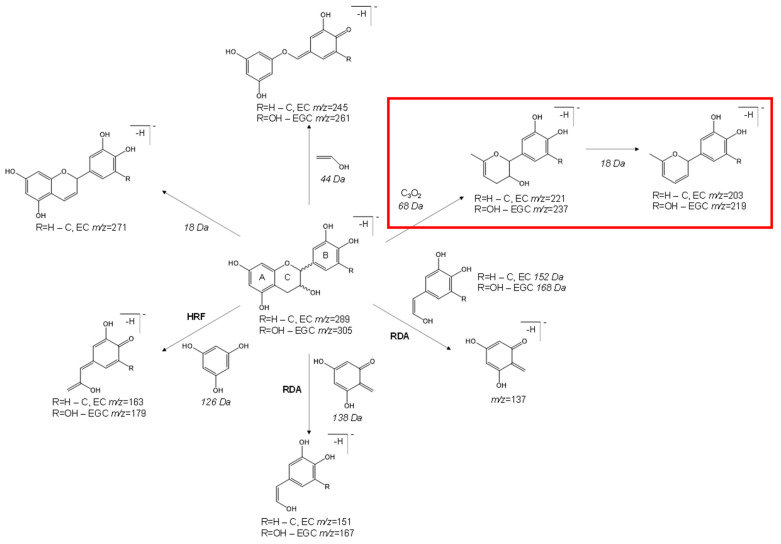
Characteristic fragmentation pathways of monomeric flavan-3-ols.  The loss of C_3_O_2_ and the subsequent losses of C_3_O_2_ and H_2_O are highlighted by the red rectangle.  RDA—retro-Diels–Alder, HRF—heterocyclic ring fission, and loss of water molecule [36,37,38,39].

The method development began with studying the fragmentation patterns of the various monomer standards, including catechin, epicatechin, epicatechin gallate, and epigallocatechin gallate, as well as the dimeric procyanidin B1 and B2, and the trimeric procyanidin C1. This analysis aimed to select the ions for quantification and qualification. The product ion mass spectra obtained from the chosen ions are shown in Figure 1, and the selected ions are presented in Table 3. In the negative ion mass spectrum (MS) of catechin, the (M − H)^−^ ion with *m*/*z* 289 can be observed. The MS/MS spectrum of the *m*/*z* 289 ion (Figure 1A) revealed major product ions with *m*/*z* 271, 245, 221, 203, 163, 151, and 137. The ion with *m*/*z* 271 resulted from the loss of water (18 Da), while the ion with *m*/*z* 245 resulted from the loss of ethenol (H_2_C=CH-OH, 44 Da). The ion with *m*/*z* 163 (M – H − 126 Da) may have formed after the elimination of ring A from catechin through heterocyclic ring fission (HRF) (Scheme 1 [54,55,56]). The ion with *m*/*z* 137 may have resulted from a retro-Diels–Alder (RDA) fission of ring C (Scheme 1). Likewise, the ion at *m*/*z* 151 can be explained by the loss of 138 Da, which was attributed to the same mechanism. The presence of the two ions at *m*/*z* 221 and 203 in the MS/MS spectra of catechin is uncommon but can be explained by the loss of C_3_O_2_ and consecutive losses of C_3_O_2_ and H_2_O (Scheme 1). This mechanism has been described for other flavonoids [2,56]. It is noteworthy that these fragment ions are not normally described in the negative MS/MS spectra of catechin, epicatechin, gallocatechin, and epigallocatechin [36,37,38,39]. However, these ions are also observed in the MS/MS spectra of epicatechin (Figure 1B) and epigallocatechin (with fragment ions at *m*/*z* 237 and 219, Figure 1C). The fragmentation patterns of (+)-catechin and (−)-epicatechin were identical (Figure 1A,B), indicating that the configuration at C3 does not influence the fragmentation of flavan-3-ols [36]. Similarly, the same fragmentation pattern was observed for epigallocatechin, although the ion containing the C-ring exhibited an *m*/*z* value that was 16 Da higher compared to catechin and epicatechin. Consequently, fragment ions at *m*/*z* 261, 237, 219, 179, and 167 were observed (Figure 1C).

For epicatechin gallate (*m*/*z* 441), the MS/MS product ion spectrum showed two ions that specifically indicated the presence of an unmodified galloyl ester function. These ions at *m*/*z* 289 (Figure 1D) represent the [M − H − 152]^−^ ion where the ester function was lost as a neutral species, and the *m*/*z* 169 ion, indicating the presence of an unmodified and intact gallic acid anion. The same fragmentation pattern was observed for epigallocatechin gallate (*m*/*z* 457), with the ions at *m*/*z* 305 and *m*/*z* 169 (Figure 1E). This fragmentation pattern aligns with the findings reported in the literature [57].

The negative ion mass spectrum (MS1) of procyanidin B1 showed the [M − H]^−^ ion with *m*/*z* 577. The MS/MS spectrum of the ion with *m*/*z* 577 (Figure 1F) showed major product ions, with *m*/*z* 456 attributed to the HRF with a loss of 129 Da. The fragment ion at *m*/*z* 425 was formed by RDA fragmentation (−152 Da), which, upon loss of water, yielded the fragment ion at *m*/*z* 407. The fragment ion at *m*/*z* 273 can be explained by the RDA of fragment ion at *m*/*z* 425. The fragment ion at *m*/*z* 299 can be attributed to the RDA fragmentation of fragment ion at *m*/*z* 451. The fragment ion at *m*/*z* 289 was formed through quinone methide fragmentation (QM) of the parent ion, and the ion at *m*/*z* 245 was formed by the loss of ethenol from the fragment ion at *m*/*z* 289. The same fragmentation pattern was observed for procyanidin B2 (Figure 1G). These fragmentation patterns are consistent with the findings reported in the literature [36,37,38].

The trimer C1 ([M − H]^−^ of *m*/*z* 865) exhibited the same fragmentation pattern as described for the dimers (Figure 1H), with fragment ions attributed to the HRF fragmentation (*m*/*z* 739/451), RDA fragmentation (*m*/*z* 713/425), RDA fragmentation followed by the loss of water (*m*/*z* 695/407), HFR fragmentation followed by RDA fragmentation (*m*/*z* 587/299), RDA fragmentation followed by another RDA fragmentation (*m*/*z* 561/273), QM fragmentation (*m*/*z* 577/287), and QM fragmentation followed by the loss of ethenol (*m*/*z* 533/245) (Table 3). In the negative product ion tandem mass spectrum of procyanidin C1 shown in Figure 1H, the dimer doublet ions of *m*/*z* 575 and 577 and monomer doublet ions of *m*/*z* 287 and 289 showed the 2 amu mass difference characteristic of quinone methide formation by B-type procyanidins [37].

To optimize method sensitivity and selectivity, neutral losses resulting in the most abundant ions and fragment ions with higher *m*/*z* values were selected and are highlighted in bold in Table 3.

#### 3.1.2. Development of Analysis Conditions and Comprehensive Sample Preparation

**Figure 2:**
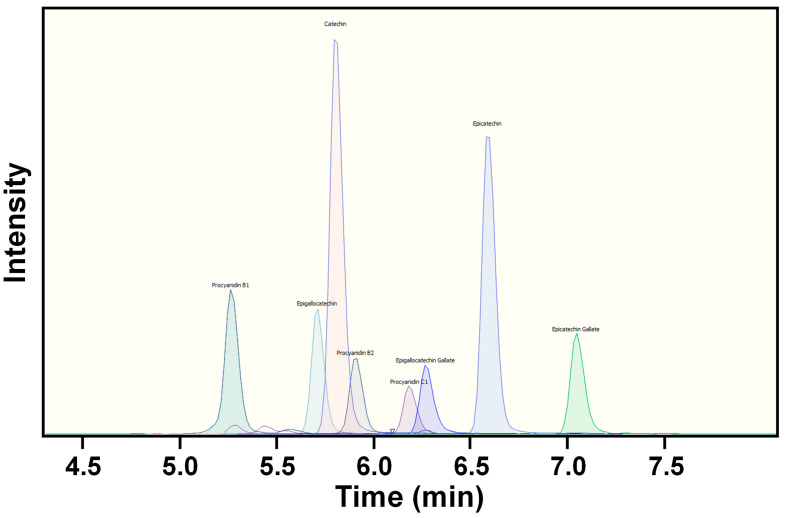
UPLC–Q-TOF MS/MS total ion chromatogram of selected ions of monomeric, dimeric, and trimeric flavan-3-ols standards described in Table 1 after broadband collision-induced dissociation (bbCID).

HPLC was utilized to separate monomeric, dimeric, and trimeric proanthocyanidins in wine using a reversed-phase C18 column. Various factors such as mobile phase composition, column temperature, column dimension, flow rate, injection volume, and binary gradient parameters were assessed. It is common to observe signal suppression caused by co-eluting matrix components. Similarly, later-eluting proanthocyanidin peaks can be overestimated due to the accumulation of charged apolar compounds [58]. To mitigate the ion suppression phenomena, wine samples were progressively diluted, and a dilution of 1:20 was determined to be the optimal choice according to the spike and recovery results (further discussed below). Additionally, MS detection allows for signal overlap, resulting in shorter run times without compromising analytical performance, as broadband fragmentation was employed to obtain fragment ions of all parent ions [38]. Under these conditions, a flow rate of 0.3 mL/min and a gradient elution time of 10 min were used. A typical chromatogram is shown in Figure 2. When compared to LC–MS methods described in the literature for wine analysis, the sample handling is simpler. For example, González-Manzano et al. [59] performed the separation of wine proanthocyanidins by SPE using an Oasis MCX cartridge and further analysis of wine proanthocyanidins by RP-HPLC–DAD-ESI-MS using an ion-trap mass analyzer with a chromatographic run of 60 min. Teixeira et al. [60] described a reversed-phase LC–EI-MS method, with an ion trap analyzer, involving extensive sample preparation, including ethyl acetate for PA isolation and fractionation by gel chromatography with TSK Toyopearl HW40(s), using a chromatographic run time of 95 min. On the other hand, Vrhovsek et al. [61] described UPLC–MS/MS using a triple quadrupole for targeted analysis of multiple classes of phenolic compounds in wine with simple filtration of wine and a 15-min chromatographic run.

During the recovery experiments, an interesting observation was made. When a mixture containing all the standards was added to the wine, it was noticed that the area of certain proanthocyanidin peaks experienced a significant increase after repeated sample injections. In some instances, the area even doubled. This effect was attributed to the presence of oxidized proanthocyanidins in the quinone form in the wine, which were subsequently reduced, resulting in recovery values that exceeded 100%. To tackle this problem, a solution was implemented by adding ascorbic acid to the samples at a final concentration of 5 g/L. The addition of ascorbic acid resulted in a prompt increase in the area of certain proanthocyanidins, which varied among different wines. This increase was attributed to the reduction of pre-existing oxidized proanthocyanidins in the wine. Furthermore, the inclusion of ascorbic acid assisted in preserving the area of the proanthocyanidins in the sample for at least one week when stored at room temperature.

### 3.2. Method Validation

The method’s performance characteristics were evaluated concerning the linear range, detection and quantification limits, repeatability, intermediate precision expressed as within-day and between-day relative standard deviations, and recovery. These values are listed in Table 4.

**Figure 3:**
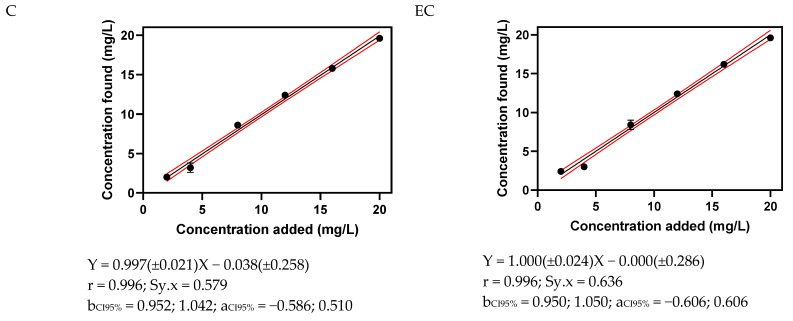

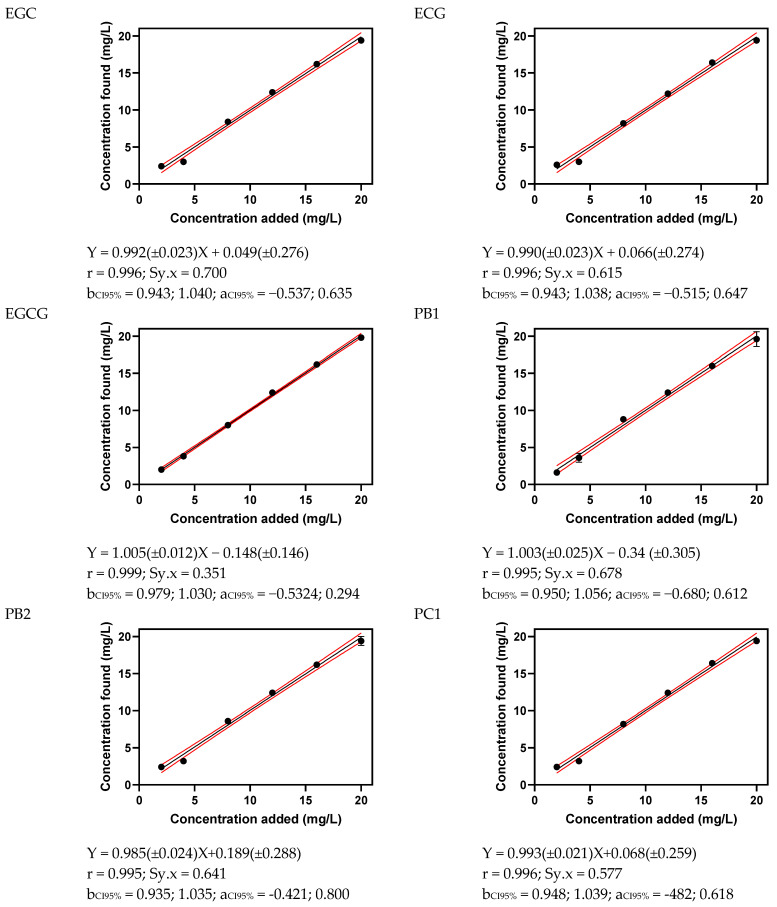
Recovery results for the flavan-3-ols, as well as dimeric and trimeric procyanidins, from the spike and recovery experiments from 2.0 mg/L to 20 mg/L (n = 6 × 3). •—Average recovery values. Error bars—standard deviation; ꟷ—best line fit, ꟷ—95% confidence intervals of the best line fit.

The quality coefficient (QCmean) was utilized to assess the calibration line’s quality [50]. The QCmean for the PA calibration curves ranged from 3.38% for epicatechin to 7.54% for procyanidin C1 (Table 4). Additionally, the linearity of the regression lines was evaluated using the Mandel’s fitting test [51]. The linear quadratic model for the regression lines did not differ significantly (*p* < 0.05) from the linear model in the 0.1 to 1 mg/L concentration range, indicating that all calibration curves passed the linearity test. The proposed method exhibited detection limits (LOD) ranging from 0.06 and 0.11 mg/L and quantification limits (LOQ) ranging from 0.19 to 0.32 mg/L (Table 4). Within-day repeatability ranged from 2.17% to 7.34% and between-day repeatability ranged from 3.07% to 13.05%. These values comply with the acceptance criterion of RSD values below 20%. For repeatability conditions, acceptable values fell within the range of 0.3 to 1.3 according to the Horwitz criteria (HorRat) [62,63]. Method accuracy was determined through spike and recovery experiments ranging from 2.0 mg/L to 20 mg/L on a red wine. Figure 3 shows the very good recoveries achieved for all flavan-3-ols, as well as dimeric and trimeric procyanidins, with recovery values ranging from 98.5% for procyanidin B2 to 100.5% for catechin. None of the slopes significantly deviated from 1 (*p* < 0.05).

**Figure 4:**
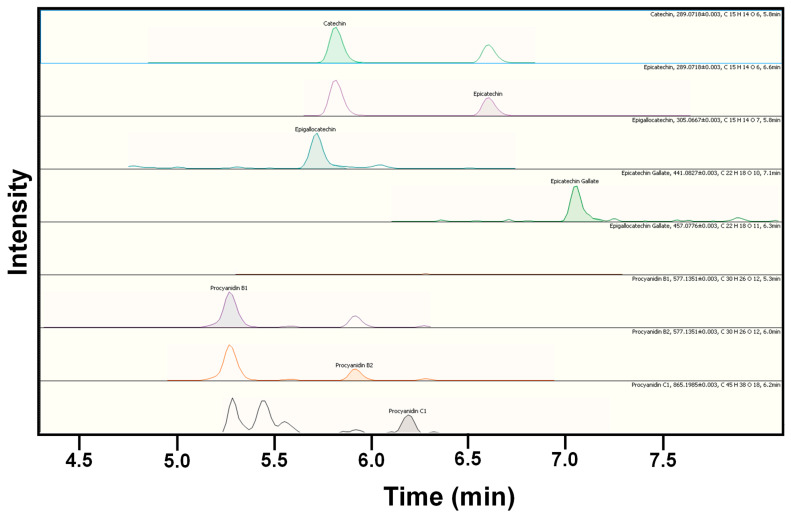
UPLC–Q-TOF MS/MS total ion chromatogram of selected ions of monomeric, dimeric, and trimeric flavan-3-ols described in Table 1 after broadband collision-induced dissociation (bbCID) of wine 9.

Table 5 presents the concentrations (mg/L) of these eight compounds in nine different red wine samples. Samples 1 to 6 are red wines from the Douro region, while samples 7 to 9 are red wines from the Alentejo region. Figure 4 shows a typical chromatogram obtained for wine 9. The values displayed represent the average of three measurements.

Significant variability was observed in the levels of monomeric flavan-3-ols and oligomeric PAs within wines from the same region, and no significant differences were found between wines from the two regions. Catechin and epicatechin concentrations ranged from 9.5 mg/L to 27.7 mg/L and from 5.2 mg/L to 22.4 mg/L, respectively. For the dimers, procyanidin B1 and B2, the values ranged from 4.6 mg/L to 42 mg/L and from <LOQ to 20.7 mg/L, respectively. The values for the trimer, procyanidin C1, ranged from <LOQ to 7.0 mg/L. Wine 8 from Alentejo presented the highest levels of both monomeric and oligomeric PAs, totaling 120.8 mg/L (Table 5). Comparing our data with findings reported in the literature is challenging due to the significant impact of grape variety and climatic conditions on wine catechin concentration [64]. However, in general, our results are on the same order of magnitude as those obtained in previous studies [41,65,66,67,68,69,70,71]. These findings demonstrate the suitability of the method for its intended use.

### 3.3. Evaluation of the Use of Patatin, Pea Protein, and Gelatin for Removing Monomeric Flavan-3-ols and Dimeric and Trimeric Procyanidins

**Figure 5:**
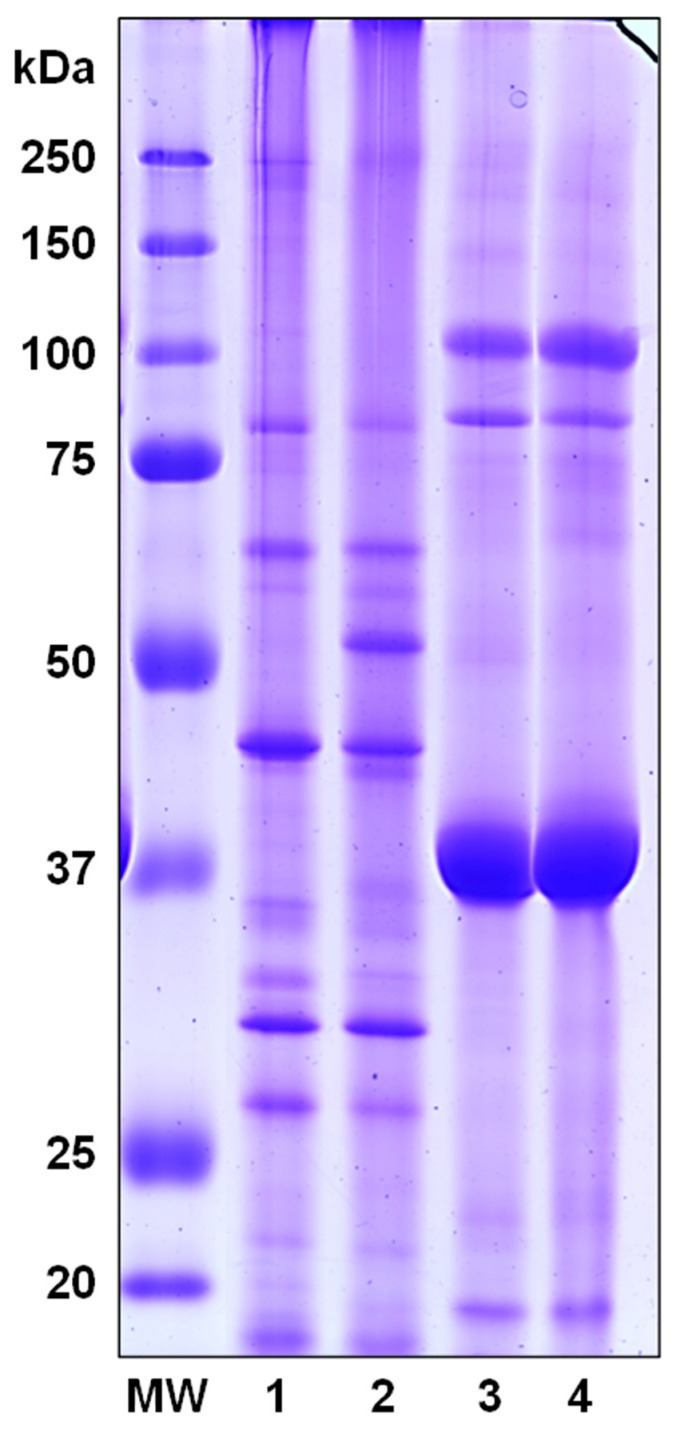
SDS-PAGE profile of the protein fining agents. Lane 1, pea protein 1; lane 2, pea protein 2; lane 3, patatin 1; lane 4, patatin 2. Sizes (in kilodaltons) of protein molecular weight markers are shown on the left. MW—molecular weight (sizes of protein markers in kilodaltons; Precision Plus Protein™ All Blue Prestained Protein Standards, Bio-Rad, CA, USA).

For evaluating the efficiency of patatin and pea protein compared to gelatin in removing potentially bitter monomeric, dimeric, and trimeric flavan-3-ols, two patatin and two pea protein preparations available on the market were used. To compare their protein composition profiles, they were analyzed using sodium dodecyl sulfate polyacrylamide gel electrophoresis (SDS-PAGE, Figure 5).

Regarding the electrophoretic profile of commercially obtained pea proteins, differences in intensity were observed for certain protein bands. Specifically, variations were noted in the band slightly above 75 kDa, between 50 kDa and 75 kDa, between 37 kDa and 50 kDa, and slightly above 25 kDa, with the highest intensity consistently observed for the protein from pea 1 (Lane 1, Figure 5). Notably, a protein band slightly above 50 kDa was present in pea 2 (Lane 2, Figure 5), which was absent in pea 1, along with other minor differences in the absence/presence slightly below 37 kDa. Both pea protein preparations exhibited distinct and representative bands, which appeared in decreasing order of molecular weights: slightly above 75 kDa, between 37 kDa and 75 kDa, and between 25 kDa and 37 kDa. These findings align with previous research by other authors, who also associated the band slightly above 75 kDa and the range between 50 kDa and 75 kDa with convicilin subunits. Furthermore, the band slightly above 50 kDa (approximately 60 kDa) was attributed to legumin monomers derived from the native hexameric form of legumins, which underwent SDS-induced reduction to form monomers. Additionally, several other bands were observed with approximate weights of 18 kDa, 34 kDa, and 50 kDa, indicating the presence of dissociated vicilin trimers [72].

The electrophoretic profile of the two commercial patatin samples was highly similar, with minor differences mainly in the intensity of certain protein bands, particularly in the molecular weight range of 50 kDa to 75 kDa. Patatin 2 (Lane 4, Figure 5) exhibited higher intensity in some bands within this range. Additionally, slight variations were observed in the electrophoretic profile below 25 kDa and above 20 kDa. In patatin 1 (Lane 3, Figure 5), a band was present within the intermediate range of this molecular weight, while it was absent in patatin 2. Overall, both samples showed proteins spanning the entire range of molecular weights analyzed, ranging from slightly below 20 kDa to 250 kDa. The most notable proteins could be observed in descending order, with two bands between 75 and 100 kDa, a prominent band at 37 kDa, and another around 20 kDa, which, although less intense, was still representative. These findings align with previous studies, particularly regarding the presence of the prominent band above 37 kDa, which was attributed to patatin. Patatin is widely recognized as the major component of potato protein isolate, constituting approximately 40% of its composition. Additionally, proteins above 75 kDa up to 250 kDa, and even higher molecular weight were observed, which could be attributed to the high-molecular-weight fractions, and protease inhibitors below 25 kDa [73].

Two different wine matrices were utilized for the fining experiments. Patatin and pea protein were applied at doses ranging from 5 g/L to 30 g/L, following the limits set by the OIV [74], and gelatin was applied at the same doses. The results obtained are shown in Table 6 and Table 7. The developed LC–MS method was used to quantify monomeric and oligomeric PAs in untreated and treated red wines with different fining agents. However, even at the highest doses of patatin, pea protein, and gelatin, no removal of flavan-3-ol, dimeric, or trimeric PAs was achieved, as concentrations of flavan-3-ols and PAs in fined wines were not significantly different from control wine (Table 6 and Table 7). This indicates their inefficiency for this purpose in these two wine matrices.

Similarly, Jourdes et al. [75], after applying a range of animal and vegetable protein fining agents and mixtures of these protein fining agents with PVPP or bentonite, found that fining agents without PVPP were not capable of precipitating monomeric or dimeric condensed tannins. Another study examined the impact of alternative protein fining agents, including patatin and pea protein, on the phenolic composition of Syrah red wines at two different stages (2 months (W2) and 12 months (W12) from the end of fermentation). The authors obtained contradictory results in the two wine matrices analyzed. For the wine aged for 12 months (W12), both patatin and pea protein showed inefficiency in reducing levels of procyanidin B1, procyanidin B2, procyanidin B2 3-*O*-gallate, procyanidin B7, epicatechin gallate, and some oligomeric proanthocyanidins. A reduction in catechin and epicatechin levels was noted for pea protein treatment, and a reduction in only catechin was noted for patatin treatment. On the other hand, for the youngest wine (W2), the authors found that patatin demonstrated a greater ability to reduce the levels of monomeric (catechin and epicatechin), dimeric (procyanidin B1, procyanidin B2, procyanidin B2 3-*O*-gallate), and some oligomeric proanthocyanidins compared to pea protein. Pea protein exhibited the lowest efficiency in reducing these compounds, showing a modest decrease in some dimeric and oligomeric flavan-3-ols. However, both patatin and pea protein were unable to reduce the content of procyanidin B7, epicatechin gallate, and some oligomeric proanthocyanidins [76]. Other studies have also reported a low efficiency of native pea protein in removing flavanols. When compared to gelatin, it consistently exhibited significantly lower efficacy in reducing the levels of catechin, epicatechin, and proanthocyanidin dimers and trimers. Interestingly, when the authors hydrolyzed the pea protein using a trypsin-like protease, it demonstrated a much higher capacity to interact with oligomeric procyanidins compared to the native proteins. This enzymatically treated pea protein led to a reduction ranging from 30% (for monomeric species) to more than 70% (for dimers and trimers). The observed differences are likely attributed to variations in the molecular size, hydrophobicity, and conformation of pea proteins and their hydrolytic fragments [28].

Nevertheless, Río Segade et al. [77], highlighted that both the effectiveness of vegetal-derived proteins and the target compounds are influenced by the phenolic composition of the wine. According to Granato et al. [78] polymeric PAs are significantly reduced by a higher number of vegetal fining agents compared to monomeric and oligomeric PAs, possibly because they are precipitated quickly. The higher number of phenolic rings present in more polymerized PAs increases their hydrophobicity, facilitating flavanol–protein interactions. However, the formation of insoluble protein–PA complexes is not solely influenced by hydrophobic forces but involves a combination of multiple interactions with distinct thermodynamic and kinetic characteristics [78,79]. Regarding this matter, it is acknowledged that commercially available protein preparations undergo denaturation and aggregation during processing. These factors may limit the effectiveness of these proteins in reducing flavanol levels. Furthermore, the characteristics of the red wine matrices themselves could also play a role in explaining our observed results.

Fining agents derived from vegetable or animal proteins can yield varying results, depending on their origin, dosage, and the phenolic profile and composition of the wine. Therefore, it is advisable to perform preliminary trials for wine fining, along with instrumental measurements, to achieve optimal outcomes.

## 4. Conclusions

A simple, fast, and selective UPLC–Q-TOF MS/MS broadband collision-induced dissociation MRM method was developed, optimized, and validated for the determination of monomeric, dimeric, and trimeric flavan-3-ols in red wines. The method involved sample dilution, sample stabilization with ascorbic acid, and a 10 min chromatographic run. The addition of ascorbic acid (5 g/L) in sample preparation improved the accuracy and precision of the determination by enhancing sample stability. With the MRM detection providing high selectivity, the method achieved good precision (repeatability between 2.2 to 7.3%), accuracy (recovery between 98.5 to 100.5%), and detection (0.3 mg/L to 0.7 mg/L) and quantification (0.8 mg/L to 2.2 mg/L) limits. The proposed method was applied with success to nine different wine matrices without any issues.

The developed method was then employed to evaluate the efficiency of patatin, pea protein, and gelatin in removing these bitter tasting flavan-3-ols from red wines. However, none of the three protein fining agents were effective in removing these bitter compounds in the two different wine matrices tested. Therefore, it is highly recommended to conduct preliminary trials specific to the wine matrix and measure the actual efficiency to ensure optimal outcomes.

## Figures and Tables

**Table 1:** Elution program.

Flow (mL/min)	Retention (min)	%A	%B
0.3	0.0	95	5
0.3	2.0	95	5
0.3	10.0	20	80
0.3	10.0	1	99
0.3	14.0	1	99
0.3	14.0	95	5
0.3	16.5	95	5

**Table 2:** Main enological characteristics of the wine samples.

	Wine 1	Wine 2
Alcohol content (% *v/v*)	14.2	14.3
Specific gravity (20 °C) (g/cm^3^)	0.9929	0.9935
Titratable acidity (g/L tartaric acid)	5.8	6.3
Volatile acidity (g/L acetic acid)	0.69	0.86
pH	3.50	3.80

**Table 3:** Retention time (Rt) and ESI/Q-TOF tandem mass product ions (*m*/*z*) of flavan-3-ol monomers and B-type dimers and trimers.

	Rt	[M − H]^−^	H_2_O	C_2_H_4_O	C_3_O_2_	C_3_O_2_/H_2_O			HRF	RDA	RDA	RDA/H_2_O	HRF/RDA	RDA/RDA	QM	QM/C_2_H_4_O
C	5.85	289	271 (18) ^a^	**245** (44)	**221** (68)	**203** (86)			163 (126)	151 (138)	137 (152)					
EC	6.64	289	271 (18)	**245** (44)	**221** (68)	**203** (86)			163 (126)	151 (138)	137 (152)					
EGC	5.75	305	-	**261** (44)	**237** (68)	**219** (86)			179 (126)	167 (138)	137 (168)					
ECG	7.09	441					**289** (152)	**169** (272)		303 (138)						
EGCG	6.30	457					**305** (152)	**169** (285)								
B1	5.31	577							**451** (126)		**425** (152)	407 (18)	299 (152)	273 (152)	**289**	245 (44)
B2	5.95	577							**451** (126)		**425** (152)	407 (18)	299 (152)	273 (152)	**289**	245 (44)
C1	6.22	865							**739** (126) 451 (126)		**713** (152) 425 (152)	695 (18) 407 (18)	587 (152) 299 (152)	561 (152) 273 (152) *	**577** 287	533 (44) 245 (44)

^a^ Neutral losses are shown in parentheses. Abbreviations: C—catechin, EC—epicatechin, EGC—epigallocatechin, ECG—epicatechin-3-*O*-gallate, EGCG—epigallocatechin-3-*O*-gallate, B1—procyanidin B1, B2—procyanidin B2, C1—procyanidin C1. *After the first QM fragmentation of the trimer. Selected ions for qualification and quantification are presented in bold.

**Table 4:** Validation parameters of the UPLC–MS method developed for the analysis of monomeric, dimeric, and trimeric proanthocyanidins.

Compounds	R^2^	LOD (mg/L)	LOQ (mg/L)	QC_mean_ (%)	Repeatability (% RSD_r_)	Intermediate Repeatability (%RSD_R_)
C	0.9939	0.5	1.5	3.65	7.34	10.98
EC	0.9922	0.7	2. 0	3.38	5.23	13.05
ECG	0.9904	0.6	1.8	7.34	4.46	3.07
EGC	0.9909	0.3	0.8	6.66	4.20	9.36
EGCG	0.9968	0.6	1.7	3.41	4.07	4.50
B1	0.9924	0.5	1.6	3.84	4.88	3.96
B2	0.9916	0.5	1.5	5.27	2.17	3.24
C1	0.9921	0.7	2.2	7.54	3.57	8.59

Abbreviations: C—catechin, EC—epicatechin, ECG—epicatechin gallate, EGC—epigallocatechin: EGCG—epigallocatechin gallate, B1—procyanidin B1: B2—procyanidin B2, C1—procyanidin C1, LOD—limit of detection, LOQ—limit of quantification.

**Table 5:** Concentrations (mg/L) of selected proanthocyanidins and monomers obtained using the UPLC–MS method developed.

Sample	C	EC	ECG	EGC	EGCG	B1	B2	C1
1	19.3 ± 2.1	10.1 ± 1.3	<LOQ	<LOQ	<LOQ	39.9 ± 1.6	15.3 ± 0.5	6.3 ± 0.5
2	16.9 ± 1.9	9.7 ± 1.3	<LOQ	<LOQ	<LOQ	38.9 ± 1.5	14.6 ± 0.5	4.9 ± 0.4
3	20.0 ± 2.2	10.6 ± 1.4	<LOQ	<LOQ	<LOQ	36.6 ± 1.4	13.4 ± 0.4	4.7 ± 0.4
4	12.3 ± 1.4	7.7 ± 1.0	<LOQ	<LOQ	<LOQ	31.2 ± 1.2	12.3 ± 0.4	3.8 ± 0.3
5	11.2 ± 1.2	5.2 ± 0.7	<LOQ	<LOQ	<LOQ	20.5 ± 0.8	6.5 ± 0.2	<LOQ
6	9.5 ± 1.0	4.0 ± 0.5	<LOQ	<LOQ	<LOQ	4.6 ± 0.2	<LOQ	<LOQ
7	17.4 ± 1.9	9.9 ± 1.3	<LOQ	<LOQ	<LOQ	21.7 ± 0.9	7.7 ± 0.3	<LOQ
8	27.7 ± 3.0	22.4 ± 2.9	<LOQ	<LOQ	<LOQ	42.0 ± 1.7	20.7 ± 0.7	7.0 ± 0.6
9	14.1 ± 1.5	8.3 ± 1.1	<LOQ	<LOQ	<LOQ	26.9 ± 1.1	10.7 ± 0.3	2.9 ± 0.2

Abbreviations: C—catechin, EC—epicatechin, ECG—epicatechin gallate, EGC—epigallocatechin: EGCG—epigallocatechin gallate, B1—procyanidin B1: B2—procyanidin B2, C1—procyanidin C1, LOQ—limit of quantification.

**Table 6:** Analytical results for proanthocyanidins in the study by LC–MS of the unfined red wine from Alentejo’s region and the same wine after different fining treatments (mean ± SD).

Fining Agents	Monomers (mg/L)		Dimers (mg/L)		Trimer (mg/L)	Gallates (mg/L)		
	CAT	EPI	B1	B2	C1	EPI GAL	EPIGAL	EPIGAL GAL
T	14.1 ± 0.7	9.5 ± 0.4	8.9 ± 0.5	3.7 ± 0.3	<LOQ	<LOQ	<LOQ	<LOQ
G5	15.3 ± 0.5	10.5 ± 0.2	10.2 ± 0.3	4.5 ± 0.2	<LOQ	<LOQ	<LOQ	<LOQ
G15	15.7 ± 0.7	10.5 ± 0.1	10.6 ± 0.3	4.4 ± 0.1	<LOQ	<LOQ	<LOQ	<LOQ
G30	15.7 ± 0.5	10.4 ± 0.3	10.8 ± 0.2	4.4 ± 0.1	<LOQ	<LOQ	<LOQ	<LOQ
P_1_5	16.2 ± 0.3	10.6 ± 0.4	11.1 ± 0.2	4.4 ± 0.2	<LOQ	<LOQ	<LOQ	<LOQ
P_1_15	16.7 ± 0.1	10.9 ± 0.2	11.3 ± 0.1	4.5 ± 0.0	<LOQ	<LOQ	<LOQ	<LOQ
P_1_30	16.1 ± 0.7	10.7 ± 0.2	11.3 ± 0.1	4.5 ± 0.2	<LOQ	<LOQ	<LOQ	<LOQ
P_2_5	16.3 ± 0.2	10.6 ± 0.3	11.4 ± 0.1	4.6 ± 0.0	<LOQ	<LOQ	<LOQ	<LOQ
P_2_15	16.7 ± 0.1	10.7 ± 0.1	11.5 ± 0.2	4.7 ± 0.0	<LOQ	<LOQ	<LOQ	<LOQ
P_2_30	16.6 ± 0.3	10.7 ± 0.1	11.6 ± 0.1	4.6 ± 0.0	<LOQ	<LOQ	<LOQ	<LOQ
PP_1_5	17.1 ± 0.1	10.9 ± 0.2	11.7 ± 0.1	4.7 ± 0.1	<LOQ	<LOQ	<LOQ	<LOQ
PP_1_15	17.2 ± 0.2	10.9 ± 0.1	12.0 ± 0.1	4.8 ± 0.1	<LOQ	<LOQ	<LOQ	<LOQ
PP_1_30	16.9 ± 0.3	11.0 ± 0.2	11.8 ± 0.1	4.9 ± 0.1	<LOQ	<LOQ	<LOQ	<LOQ
PP_2_5	17.0 ± 0.3	10.9 ± 0.1	11.8 ± 0.2	4.8 ± 0.0	<LOQ	<LOQ	<LOQ	<LOQ
PP_2_15	17.1 ± 0.3	10.9 ± 0.4	12.1 ± 0.1	4.9 ± 0.0	<LOQ	<LOQ	<LOQ	<LOQ
PP_2_30	16.8 ± 0.3	10.9 ± 0.1	12.1 ± 0.2	4.9 ± 0.0	<LOQ	<LOQ	<LOQ	<LOQ

(T) Unfined wine. (G5) Gelatin 5 g/hL. (G15) Gelatin 15 g/hL. (G30) Gelatin 30 g/hL. (P_1_5) Patatin one 5 g/hL. (P_1_15) Patatin one 15 g/hL. (P_1_30) Patatin one 30 g/hL. (P_2_5) Patatin two 5g/hL. (P_2_15) Patatin two 15 g/hL. (P_2_30) Patatin two 30 g/hL. (PP_1_5) Pea protein one 5 g/hL. (PP_1_15) Pea protein one 15 g/hL. (PP_1_30) Pea protein one 30 g/hL. (PP_2_5) Pea protein two 5 g/hL. (PP_2_15) Pea protein two 15 g/hL. (PP_2_30) Pea protein two 30 g/hL. (N.D.) not detected.

**Table 7:** Analytical results for proanthocyanidins in the study by LC–MS of the unfined red wine from Douro’s region and the same wine after different fining treatments (mean ± SD).

Fining Agents	Monomers (mg/L)		Dimers (mg/L)		Trimer (mg/L)	Gallates (mg/L)		
	CAT	EPI	B1	B2	C1	EPI GAL	EPIGAL	EPIGAL GAL
T	3.0 ± 0.0	2.5 ± 0.1	12.4 ± 0.2	2.6 ± 0.1	<LOQ	<LOQ	<LOQ	<LOQ
G5	2.9 ± 0.1	2.5 ± 0.0	11.9 ± 0.2	2.5 ± 0.1	<LOQ	<LOQ	<LOQ	<LOQ
G15	2.9 ± 0.0	2.6 ± 0.0	12.4 ± 0.2	2.5 ± 0.0	<LOQ	<LOQ	<LOQ	<LOQ
G30	3.0 ± 0.1	2.6 ± 0.1	12.8 ± 0.0	2.6 ± 0.0	<LOQ	<LOQ	<LOQ	<LOQ
P_1_5	3.0 ± 0.1	2.6 ± 0.1	12.5 ± 0.4	2.6 ± 0.1	<LOQ	<LOQ	<LOQ	<LOQ
P_1_15	3.1 ± 0.1	2.6 ± 0.1	12.9 ± 0.1	2.7 ± 0.0	<LOQ	<LOQ	<LOQ	<LOQ
P_1_30	2.9 ± 0.1	2.6 ± 0.1	12.6 ± 0.0	2.6 ± 0.1	<LOQ	<LOQ	<LOQ	<LOQ
P_2_5	2.9 ± 0.0	2.6 ± 0.0	12.3 ± 0.1	2.6 ± 0.0	<LOQ	<LOQ	<LOQ	<LOQ
P_2_15	3.0 ± 0.1	2.7 ± 0.1	12.8 ± 0.2	2.6 ± 0.1	<LOQ	<LOQ	<LOQ	<LOQ
P_2_30	3.0 ± 0.1	2.7 ± 0.0	12.6 ± 0.1	2.6 ± 0.1	<LOQ	<LOQ	<LOQ	<LOQ
PP_1_5	3.0 ± 0.6	2.6 ± 0.0	12.4 ± 0.3	2.6 ± 0.1	<LOQ	<LOQ	<LOQ	<LOQ
PP_1_15	3.0 ± 0.1	2.6 ± 0.0	12.5 ± 0.2	2.5 ± 0.1	<LOQ	<LOQ	<LOQ	<LOQ
PP_1_30	3.0 ± 0.0	2.6 ± 0.1	12.4 ± 0.3	2.6 ± 0.0	<LOQ	<LOQ	<LOQ	<LOQ
PP_2_5	3.0 ± 0.1	2.6 ± 0.0	12.6 ± 0.1	2.6 ± 0.1	<LOQ	<LOQ	<LOQ	<LOQ
PP_2_15	3.0 ± 0.0	2.6 ± 0.0	12.5 ± 0.0	2.7 ± 0.0	<LOQ	<LOQ	<LOQ	<LOQ
PP_2_30	3.0 ± 0.0	2.6 ± 0.0	12.9 ± 0.0	2.3 ± 0.0	<LOQ	<LOQ	<LOQ	<LOQ

(T) Unfined wine. (G5) Gelatin 5 g/hL. (G15) Gelatin 15 g/hL. (G30) Gelatin 30 g/hL. (P_1_5) Patatin one 5 g/hL. (P_1_15) Patatin one 15 g/hL. (P_1_30) Patatin one 30 g/hL. (P_2_5) Patatin two 5g/hL. (P_2_15) Patatin two 15 g/hL. (P_2_30) Patatin two 30 g/hL. (PP_1_5) Pea protein one 5g/hL. (PP_1_15) Pea protein one 15 g/hL. (PP_1_30) Pea protein one 30 g/hL. (PP_2_5) Pea protein two 5 g/hL. (PP_2_15) Pea protein two 15 g/hL. (PP_2_30) Pea protein two 30 g/hL. (N.D.) not detected.

## Data Availability

Data are contained within the article or supplementary materials.

## Supplementary Materials

The following supporting information can be downloaded at https://www.mdpi.com/article/10.3390/foods12173313/s1: Table S1. Main enological characteristics of the wine samples used for the determination of the concentrations (mg/L) of selected proanthocyanidins and monomers using the UPLC–MS method developed.
