# Supplementary material for: Fast and Simple UPLC–Q-TOF MS Method for Determination of Bitter Flavan-3-ols and Oligomeric Proanthocyanidins: Impact of Vegetable Protein Fining Agents on Red Wine Composition
Source: Foods. 2023 Sep 2;12(17):3313. doi: 10.3390/foods12173313 (PMC10486807; doi:10.3390/foods12173313)
Supplement: Supplementary file 1 [file foods-12-03313-s001.zip › foods-2516031-supplementary.pdf]

Supplementary Material Table S1

Table S1. Main enological characteristics of the wine samples.

	Douro						Alentejo		
	Wine 1	Wine 2	Wine 3	Wine 4	Wine 5	Wine 6	Wine 7	Wine 8	Wine 9
Alcohol content (% v/v)	12.8	15.4	13.3	14.0	13.3	14.2	14.9	14.3	15.5
Specific gravity (20 °C) (g/cm^3)	0.9920	0.9900	0.9914	0.9941	0.9955	0.9908	0.9900	0.9930	0.9918
Titratable acidity (g/L tartaric acid)	4.9	5.0	4.9	6.0	5.7	5.0	5.3	5.4	5.7
Volatile acidity (g/L acetic acid)	0.65	0.55	0.31	0.34	0.33	0.39	0.44	0.54	0.66
pH	3.82	3.74	3.81	3.61	3.75	3.61	3.46	3.50	3.60
